# Plants Coping Abiotic and Biotic Stresses: A Tale of Diligent Management

**DOI:** 10.1155/2015/754754

**Published:** 2015-01-05

**Authors:** Hatem Rouached, Sikander Pal, Shimon Rachmilevitch, Marc Libault, Lam-Son Phan Tran

**Affiliations:** ^1^Biochimie et Physiologie Moléculaire des Plantes, Unité Mixte de Recherche CNRS-INRA, Université Montpellier 2, Montpellier SupAgro, Place Viala, 34060 Montpellier, France; ^2^Department of Botany, University of Jammu, Jammu , Jammu and Kashmir 180-006, India; ^3^Jacob Blaustein Institute for Desert Research, BGU, 84990 Sede-Boqer Campus, Israel; ^4^Department of Microbiology and Plant Biology, University of Oklahoma, 770 Van Vleet Oval, Norman, OK 73019, USA; ^5^Signaling Pathway Research Unit, RIKEN Center for Sustainable Resource Science, 1-7-22 Suehiro-cho, Tsurumi-ku, Yokohama 230-0045, Japan

Plants unlike other living forms are sessile thereby facing severe biotic and abiotic stresses. Plants have evolved different efficient defence responses which thrive upon a number of intrinsic factors, such as genotypic and phenotypic constitutions and developmental circumstances, and extrinsic factors like severity and duration of the stresses. Stress management uses molecular and biochemical level controls, the competence, and speed, at which a stress signal is perceived and transmitted to generate stress signal molecules and activate stress-protective mechanisms. A well-concerted action of the plants' competence at morphological, physiological, biochemical, and molecular strata regulates numerous adaptive responses to biotic and abiotic stresses. Genetic manipulations of signalling networks have been widely used to improve plant productivity under stressful conditions. Advanced biotechnological application will enable maintaining agriculture in a sustainable manner. In this special issue, we present two reviews and four research papers which address genomic, molecular, and physiological regulations as well as signalling networks dealing with plant responses to abiotic and biotic factors.

Climate change, desertification, and the rise in human population have put a severe load on agriculture and are deteriorating crop productivity. In recent years, numerous molecular and metabolic pathways involved in plant responses and adaptation to various types of environmental stresses have been identified and reported. Among hundreds of metabolic pathways identified, the role of polyamines in stress management to enhance plant acclimation and adaptation is emerging rapidly. In this special issue, a timely review by P. Rangan et al. (2014) summarizes our knowledge on biosynthesis and catabolism of polyamines and highlights recent progress in elucidating the functions of polyamines in regulation of plant responses to abiotic stresses. Given a huge genetic variation among plant species, the authors also discuss a systematic approach based on polyamine-mediated enhancement of stress tolerance which might be used as a potential strategy for screening and identification of natural variants within existing crop species. The identified genotypes that possess compatible allelic variants could be then used for the improvement of stress tolerance.

Plant-microbe interactions are at the core of symbiotic, parasitic, or mutualistic plant-microbe relationships. These interactions have displayed a unique way of mutualistic communications for a resource sharing. To shed light on the topic, a detailed review of M. Libault (2014) explains the unique mechanisms in using legumes to interact with bacteria. Leguminous plants have developed a mutualistic symbiotic relationship with rhizobium (a type of soil bacteria). Upon bacterial infection, a new root organ called nodule is developed that enables the leguminous plants to access a steady source of nitrogen through the fixation and assimilation of the atmospheric N_2_ by the symbiotic bacteria. In return, the bacteria also get benefit from the symbiotic plants that provide photosynthesis product to bacteria as source of carbon. Environmental stress or climate change, which influences the concentration of the atmospheric carbon dioxide (CO_2_), will have a significant impact on plant photosynthesis. As a consequence, this will affect the nitrogen and carbon metabolism, leading to altered nitrogen fixation efficiency. The key regulatory mechanisms controlling carbon/nitrogen balances with particular attention to legume nodulation are reviewed by coeditor M. Libault in his review article. In addition, readers can also get an overview about the effect of the change in CO_2_ level on nitrogen fixation efficiency through this review, giving rise to idea as to how we could mitigate the impact of the change in atmospheric CO_2_ concentration.

Besides the change in atmospheric CO_2_ level, water deficit is one of the major constraints for nodulation, and, as a consequence for plant productivity. This topical issue is presented here by a research article of S. Sulieman et al. (2014). In their study, the authors assessed the growth and nodulation attributes of two soybean varieties DT2008 and Williams 82 (W82), which have contrasting drought-tolerant capacity, in a symbiotic association with* Bradyrhizobium japonicum* under drought and subsequent rehydration. The authors aimed to understand the correlation between N_2_ fixation efficiency and differential drought-responsive phenotypes of DT2008 and W82. Their results also provide genetic resources and basis foundation for further genomic research that would lead to better understanding of mechanisms involved in regulation of N_2_ fixation in soybean under drought at molecular level.

Heavy metal pollution has been a matter of grave concern. Until recently, efforts have been mainly restricted to phytoremediation of soils using plant species with high metal uptake capacity such as* Brassica* species. Chromium (Cr) is a highly phytotoxic heavy metal affecting crop productivity and human health via entering the food chain. Phytoremediation of Cr-polluted soils has been mostly demonstrated in using herbaceous plants, whereas use of cotton cultivars in Cr phytoremediation is least explored. In the present issue, M. K. Daud et al. (2014) have shown the potentials of two transgenic cotton cultivars (J208 and Z905) and their hybrid line (ZD14) in Cr phytoremediation using a multiple biomarker approach. Their work showed that these cotton cultivars and hybrid line could effectively uptake and sequestrate Cr in dead parts of the plants, such as vacuole and cell wall, besides having a more highly accelerated antioxidant system. This study thus proposes a new role of cotton cultivars in phytoremediation of Cr-polluted soils.

The interactions between macro- and micronutrient homeostases have been reported in many physiological and nutritional situations. N. Bouain et al. (2014) studied the interaction between phosphate (Pi) and zinc (Zn) homeostasis in two lettuce varieties. The results revealed that the variation in Pi and Zn supplies affects the biomass and photosynthesis as well as the dynamics of Pi transport in a contrasting manner between the two varieties, indicating a genetic basis for such physiological responses. On the basis of their results, the authors proposed a working model of how Pi and Zn signalling pathways are integrated into functional networks to control plant growth.

Salinity is a major abiotic stress worldwide claiming agriculture lands and affecting productivity. Research paper by A. Matsui et al. (2014) investigated salt stress in* Arabidopsis*. The authors reported that the tasiRNA-ARF pathway is involved in controlling floral architecture in plants under drought and high salinity. The ta-siRNA biosynthesis mutants and mutated* ARF3*-overexpressing plants showed short stamen under drought and salt stress, which hampered self-pollination. This study reports for the first time that a type of ta-siRNAs (tasiRNA-ARF) plays an important role in maintaining normal stamen growth and fertilization under drought and high salinity.

